# Annual acknowledgement of reviewers

**DOI:** 10.1186/s12881-015-0153-6

**Published:** 2015-04-22

**Authors:** Tim Sands

**Affiliations:** BioMed Central, Floor 6, 236 Gray’s Inn Road, London, WC1X 8HB UK

## Abstract

The editors of BMC Medical Genetics would like to thank all our reviewers who have contributed to the journal in Volume 15 (2014).

**Rami Abou Jamra**

Germany

**Khaled Abu-Amero**

Saudi Arabia

**David Adams**

USA

**Amegnona Agbonon**

Togo

**Nurten Akarsu**

Turkey

**Ersin Akgöllü**

Turkey

**Bassam Ali**

United Arab Emirates

**Richard Allen**

USA

**Carlos Alvarez**

USA

**Ping An**

USA

**Maria Grazia Andreassi**

Italy

**Muhammad Ansar**

Pakistan

**Minoti Apte**

Belize

**Rector Arya**

USA

**Halil Ibrahim Atasoy**

Turkey

**Tania Attie-Bitach**

France

**Kit Sing Au**

USA

**Takuya Awata**

Japan

**Julia Azimova**

Russia

**Alexis Baass**

Canada

**Hideo Baba**

Japan

**Anwar Baban**

Italy

**Albino Bacolla**

USA

**Raffaele Badolato**

Italy

**Yoshiyuki Ban**

Japan

**Sandro Banfi**

Italy

**Siddharth Banka**

United Kingdom

**Donatella Barisani**

Italy

**Sulman Basit**

Saudi Arabia

**Omer Faruk Bayrak**

Turkey

**Tara Beattie**

Canada

**Jaroslav Bendl**

Czech Republic

**Kristina Bengtsson Bostrom**

Sweden

**Conceicao Bettencourt**

United Kingdom

**Weimin Bi**

USA

**Alexander Bialasiewicz**

Qatar

**Harald Binder**

Germany

**Alan Bittles**

Australia

**Francisco J Blanco**

Spain

**Jean-Louis Blouin**

Switzerland

**Elizabeth Blue**

USA

**Carolyn Bondy**

USA

**Renato Borgatti**

Italy

**Audrey Boutron**

France

**Patrice Bouvagnet**

France

**Oliver Brandau**

Austria

**Michael Briggs**

United Kingdom

**Frank Brouwers**

Netherlands

**Claudio Bruno**

Italy

**Tomas Buchler**

Czech Republic

**Kathryn Burdon**

Australia

**Matthew Butler**

USA

**Daniela Caccamo**

Italy

**Sebastiano Calandra**

Italy

**Bert Callewaert**

Belgium

**Federico Canzian**

Germany

**Chuanhai Cao**

USA

**Jorge Capdevila**

USA

**Cynthia Cardoso**

Brazil

**Aaron Carman**

USA

**Yaron Carmi**

USA

**Melissa Carter**

Canada

**Ferran Casals**

Canada

**Elizabeth Chao**

USA

**Geetha Chittoor**

USA

**Pietro Chiurazzi**

Italy

**Yoon Shin Cho**

South Korea

**Byung Yoon Choi**

South Korea

**Murim Choi**

USA

**Hélène Choquet**

USA

**Shu-Chun Chuang**

Taiwan

**Marco Cicardi**

Italy

**Marina Colombi**

Italy

**Daniela Concolino**

Italy

**William Copeland**

USA

**Francesca Cordero**

Italy

**Holly Cukier**

USA

**Raymond Dalgleish**

United Kingdom

**Bruno Dallapiccola**

Italy

**Sumita Danda**

India

**Cesare Danesino**

Italy

**Dezso David**

Portugal

**Andrea Davies**

United Kingdom

**Andrew Davis**

Australia

**Alessandro De Luca**

Italy

**Valli De Re**

Italy

**Hans Deckmyn**

Belgium

**Amos Deinard**

USA

**Maria Lisa Dentici**

Italy

**Terry Derks**

Netherlands

**Perundurai Dhandapany**

USA

**Manuel Diaz-Rios**

Puerto Rico

**Maria Cristina Digilio**

Italy

**Johanna Distefano**

USA

**Elise Drouin**

USA

**Malgorzata Drozniewska**

Poland

**David Dyment**

Canada

**Michael Eccles**

New Zealand

**Ayman El-Hattab**

United Arab Emirates

**Hatem El-Shanti**

Qatar

**Bahadir Ercan**

Turkey

**Ebrahim Eskandari-Nasab**

Iran

**Carmen Espinós**

Spain

**Raul Estevez**

Spain

**Begoña Ezquieta**

Spain

**Tove Fall**

Sweden

**David Fardo**

USA

**Carmen Fava**

Italy

**Laura Fejerman**

USA

**José Fernández-Fernández**

Spain

**Ceres Fernandez-Rozadilla**

United Kingdom

**Giovanni Battista Ferrero**

Italy

**Jean Feunteun**

France

**Joan Fibla**

Spain

**Marco Fichera**

Italy

**Judith Field**

Australia

**Marcus Fischer**

Germany

**Ilenia Foffa**

Italy

**Jose Manuel Fragoso**

Mexico

**Lude Franke**

Netherlands

**Alexis Frazier-Wood**

USA

**Guy Froyen**

Belgium

**Ricardo Fujita**

Peru

**Frédéric Fumeron**

France

**Nobuo Fuse**

Japan

**Eija Gaily**

Finland

**Meili Gao**

China

**Peisong Gao**

USA

**Eva Garcia Galloway**

Spain

**Valentina Gatta**

Italy

**Maria Gazouli**

Greece

**Bruce Gelb**

USA

**Célia Giacheti**

Brazil

**Michael Gill**

Ireland

**Mara Giordano**

Italy

**Santhosh Girirajan**

USA

**Joseph Glessner**

USA

**Michael Gollob**

Canada

**Roser Gonzalez-Duarte**

Spain

**Consuelo González-Manchón**

Spain

**Justin Grobe**

USA

**Anna Grzywacz**

Poland

**Encarna Guillen-Navarro**

Spain

**Pierre Hainaut**

France

**Hanan Hamamy**

Switzerland

**Megumi Hara**

Japan

**Muhammad Hassan**

Pakistan

**James Hejtmancik**

USA

**Chew-Kiat Heng**

Singapore

**Concepción Hernandez**

Spain

**Stephanie Hicks**

USA

**Kenji Hirayama**

Japan

**Lynne Hocking**

United Kingdom

**Janet Hoenicka**

Spain

**Peter Holmans**

United Kingdom

**Katharina Hopp**

USA

**H Dean Hosgood**

USA

**Xuwei Hou**

China

**Jinyu Huang**

China

**Tao Huang**

USA

**Yoran Hummel**

Netherlands

**Susan Huson**

United Kingdom

**Bettina Iliopoulou**

USA

**Ituro Inoue**

Japan

**Darby Jack**

USA

**Linda Jakobsen**

Denmark

**Mohamed Jamal**

USA

**Andreas Janecke**

Austria

**Lilian Jara**

Chile

**Juraj Javor**

Slovakia

**Musharraf Jelani**

Saudi Arabia

**Emma Jenkinson**

United Kingdom

**Victor Jin**

USA

**Wendy Jones**

United Kingdom

**Lidia Karabon**

Poland

**Fiona Karet**

United Kingdom

**Ariana Kariminejad**

Iran

**Daniel Kelberman**

United Kingdom

**Maria Keller**

Germany

**Jack Kent**

USA

**Madhu Khullar**

India

**Hye-Sun Kim**

South Korea

**Ying-Chin Ko**

Taiwan

**Yuta Kochi**

Japan

**Inke König**

Germany

**Michihiro Kono**

Japan

**Christian Kratz**

Germany

**Juozas Kupcinskas**

Lithuania

**Kerstin Kutsche**

Germany

**Lilia Laadhar**

Tunisia

**Riccardo Lacchini**

Brazil

**Vasiliki Lagou**

United Kingdom

**Kent Lai**

USA

**Seema Lalani**

USA

**Allen Lamb**

USA

**Edward Lammer**

USA

**Klea Lamnissou**

Greece

**Conxi Lazaro**

Spain

**Sang Hong Lee**

Australia

**Paul Leeson**

United Kingdom

**Petra Leidinger**

Germany

**Elizabeth Leslie**

USA

**Joshua Lewis**

Australia

**Grant Lewison**

United Kingdom

**Alexander Lezhava**

Singapore

**Thomas Liehr**

Germany

**Petra Liskova**

Czech Republic

**Yu Liu**

China

**Hongliang Liu**

USA

**Kuo Liu**

China

**Margarita López-Trascasa**

Spain

**Ru-Band Lu**

Taiwan

**Hermann-Josef Lüdecke**

Germany

**Xiaofeng Lv**

China

**Jonathan Magaña**

Mexico

**Sajid Malik**

Pakistan

**Eirini Manoli**

USA

**Forbes Manson**

United Kingdom

**Ashham Mansur**

Germany

**Anna Marabotti**

Italy

**Nicholas Marini**

USA

**Fernando Marson**

Brazil

**Laura Martinez**

Mexico

**Davide Martorana**

Italy

**Céline Mascaux**

Canada

**Saber Masmoudi**

Tunisia

**Tatsuo Matsunaga**

Japan

**Hirotaka Matsuo**

Japan

**Sylvie Mazoyer**

France

**Aj Mcknight**

United Kingdom

**Terri Mcveigh**

Ireland

**Christopher Medway**

USA

**Alison Merikangas**

Ireland

**Giuseppe Merla**

Italy

**Thurman Merritt**

USA

**Ludwine Messiaen**

USA

**Luisa Mestroni**

USA

**Ingrid Meulenbelt**

Netherlands

**Zosia Miedzybrodzka**

United Kingdom

**Montserrat Mila**

Spain

**Le Min**

USA

**Parvin Mirmiran**

Iran

**Jean-Paul Misson**

Belgium

**Laura Mitchell**

USA

**Deepak Modi**

India

**Seyed Mojtaba Mohaddes Ardebili**

Iran

**Angela Morgan**

Australia

**Hiroyuki Morino**

Japan

**Takayuki Morisaki**

Japan

**Ian Morison**

New Zealand

**Amelia Morrone**

Italy

**Linda Jane Mullins**

United Kingdom

**Arasambattu Kannan Munirajan**

India

**Patricia Munroe**

United Kingdom

**Alessio Naccarati**

Italy

**Muhammad Naeem**

Pakistan

**Keisuke Nagasaki**

Japan

**Shalima Nair**

Australia

**Steven Narod**

Canada

**Giovanni Neri**

Italy

**Marcella Neri**

Italy

**Sarah May Nikkel**

Canada

**Vladyslav Nikolayevskyy**

United Kingdom

**Marco Oderda**

Italy

**Trine Baur Opstad**

Norway

**Kelly Ormond**

USA

**Nick Orr**

United Kingdom

**Alfredo Orrico**

Italy

**Masao Ota**

Japan

**Laura Ottini**

Italy

**Tayfun Ozcelik**

Turkey

**Hilal Ozdag**

Turkey

**Giacomo Maria Paganotti**

Italy

**Barry Palmer**

New Zealand

**Stephen Palmer**

Australia

**Edenir Palmero**

Brazil

**Jane Pappas**

Canada

**Sunil Parikh**

USA

**Rossella Parini**

Italy

**Sofia Pavanello**

Italy

**Guiomar Perez De Nanclares**

Spain

**Flor Maria Perez-Campo**

Spain

**Hervé Perron**

Switzerland

**Claudio Pignata**

Italy

**Hugo Pinheiro**

Portugal

**Grzegorz Placha**

Poland

**Laila Poisson**

USA

**Ulrich Preuss**

Germany

**Oliver Puk**

Germany

**Annick Raas-Rothschild**

Israel

**Sivaramakrishna Rachakonda**

Germany

**Singh Rajender**

India

**Ana Ramalhinho**

Portugal

**Gudrun Rappold**

Germany

**Frank Rauch**

Canada

**Pierre Ray**

France

**Hanna Rennert**

USA

**Helen Rizos**

Australia

**Cosmeri Rizzato**

Italy

**Sébastien Robiou Du Pont**

Canada

**Nurit Rosenberg**

Israel

**Massimiliano Rossi**

France

**Margalida Rotger**

Switzerland

**Elisa Rubino**

Italy

**Malgorzata Rydzanicz**

Poland

**Saverio Sabina**

Italy

**Christoph Saely**

Austria

**Maria Saez**

Spain

**Anavaj Sakuntabhai**

France

**Pascual Sanchez-Juan**

Spain

**Richard Sandford**

United Kingdom

**Filippo Santorelli**

Italy

**Regie Lyn Santos-Cortez**

USA

**Kapaettu Satyamoorthy**

India

**Pascale Saugier-Veber**

France

**Judy Savige**

Australia

**Anna Savoia**

Italy

**Fernando Scaglia**

USA

**Raquel Scarel-Caminaga**

Brazil

**André Scherag**

Germany

**George Schmid**

Kazakhstan

**Markus Scholz**

Germany

**Mark Seielstad**

USA

**Ali Sengul**

Turkey

**Theodoros Sergentanis**

Greece

**Marco Seri**

Italy

**Tamim Shaikh**

USA

**Aiden Shearer**

USA

**Yiping Shen**

USA

**Gabriella Silvestri**

Italy

**Carly Siskind**

USA

**Sarah Smithson**

United Kingdom

**Rafal Sobota**

USA

**Xavier Solé**

Spain

**Hugo Sousa**

Portugal

**Henriët Springelkamp**

Netherlands

**Sandesh Chakravarthy Sreenath Nagamani**

USA

**Pawel Stankiewicz**

USA

**Janina Staub**

Germany

**Daniela Steinberger**

Germany

**Alexandre Stewart**

Canada

**Heidi Stöhr**

Germany

**Meitsz Su**

Taiwan

**Masaya Sugiyama**

Japan

**Lone Sunde**

Denmark

**Gérard Tachdjian**

France

**Qizhu Tang**

China

**Toshiyasu Taniguchi**

USA

**Muhammad Tariq**

USA

**Flora Tassone**

USA

**Sean Tavtigian**

USA

**Sarah Taylor**

USA

**Sebastien Theriault**

Canada

**Bobby Thomas**

USA

**Hakan Toka**

USA

**Asli Tolun**

Turkey

**Anke Tonjes**

Germany

**Frederic Tort**

Spain

**Christine Tranchant**

France

**David-Alexandre Tregouet**

France

**Rossella Tricarico**

Italy

**Barbara Triggs-Raine**

Canada

**Tai-Chung Tseng**

Taiwan

**Gregory Tsongalis**

USA

**Kam-Wah Tsui**

USA

**Hironori Ueda**

Japan

**Reinhard Ullmann**

Germany

**Takeshi Usui**

Japan

**Christine Van Broeckhoven**

Belgium

**Esther Van De Vosse**

Netherlands

**Linda Van Der Tol**

Netherlands

**Wim Van Hul**

Belgium

**Lut Van Laer**

Belgium

**Cecilia Vecoli**

Italy

**Paolo Vezzoni**

Italy

**Ales Vicha**

Czech Republic

**Alexandre Vieira**

USA

**Mauno Vihinen**

Sweden

**Bjørn Egil Vikse**

Norway

**Tatyana Vlaykova**

Bulgaria

**Lorenz Von Seidlein**

Australia

**Konstantinos Voskarides**

Cyprus

**Veronika Vymetalkova**

Czech Republic

**Keith Walley**

Canada

**Junbai Wang**

Norway

**Tzu-Hao Wang**

Taiwan

**Kui Wang**

China

**Gannan Wang**

China

**Niels Weinhold**

Germany

**Marquitta White**

USA

**Margo Whiteford**

United Kingdom

**James Whitehorn**

United Kingdom

**Christina Willenborg**

Germany

**Judy Wong**

Canada

**Xin-Yi Xia**

China

**Chao Xing**

USA

**Kui Xu**

USA

**Raquel Yahyaoui**

Spain

**Sailu Yellaboina**

USA

**Shea Ping Yip**

China

**Ke-Da Yu**

China

**Yuri Yurov**

Russia

**Laurie Zawertailo**

Canada

**Qi Zeng**

USA

**Ling Zeng**

China

**Sen Zhang**

China

**Hua Zhao**

USA

**Qing Zheng**

USA

**Yan Zheng**

USA

**Ying Zhu**

USA

**Marcella Zollino**

Italy

